# Identification of Molecular Subtype and Prognostic Signature for Prostate Adenocarcinoma based on Neutrophil Extracellular Traps

**DOI:** 10.7150/jca.93275

**Published:** 2024-03-17

**Authors:** Yanfen Zheng, Hui Sun, Shanshan Yang, Wei Liu, Guanmin Jiang

**Affiliations:** Department of Clinical Laboratory, The Fifth Affiliated Hospital of Sun Yat-sen University, Zhuhai, China.

**Keywords:** Prostate adenocarcinoma, Neutrophil Extracellular Traps, Prognostic model, MMP9

## Abstract

**Background**: Prostate adenocarcinoma (PRAD) is one of the most common cancers in male. Increasing evidences pointed out that Neutrophil Extracellular Traps (NETs) play an important role in tumor angiogenesis, tumor metastasis and drug resistance. However, limited systematic studies regarding the role of NETs in PRAD have been performed. Identification of biomarkers based on NETs might facilitate risk stratification which help optimizing the clinical strategies.

**Methods**: NETs-related genes with differential expressions were identified between PRAD and adjacent normal tissues in TCGA-PRAD dataset. Consensus cluster analysis was performed to determine the PRAD subtypes based on the different-expressed NETs-related genes. The difference of pathway enrichment, infiltrating immune cell and genomic mutation were also evaluated between subtypes. LASSO cox regression analysis was conducted to construct a NETs-related prognostic signature.

**Result**: We identified 19 NETs related genes with differential expressions between PRAD and adjacent normal tissue in TCGA-PRAD dataset. Two significant subtypes were identified based on these 19 genes by consensus cluster analysis, namely subtype 1 and subtype 2. Significant differences in prognosis, immune infiltration and tumor mutation burden were observed in subtypes. LASSO Cox regression analysis identified a NETs-associated prognostic signature including 13 genes, and this signature had a good performance in predicting the progression-free survival of PRAD patients. Further integrated analysis indicated that MMP9 mostly expressed in Mono/Macrophage cells might play a role in regulating NETs formation via neutrophil activation in PRAD.

**Conclusion**: To sum up, the current study identified two NETs-related molecular subtypes and based on which constructed a prognostic signature for PRAD.

## Introduction

Prostate adenocarcinoma is a leading cause of morbidity and mortality in male, affecting millions of men globally 1, 2. It is estimated that there were 288,300 new cases and 34,700 cases of death caused by PRAD in the US in 2023 3. In China, it is estimated that there were 78,300 cases of PRAD in 2016. The age standardized incidence rate reached 6.72/10,000, which increased significantly 4. As higher risk for PRAD is observed above the age of 70 years 5, the disease burden brought by PRAD will increase with aging population. For localized PRAD, most patients will receive surgery and adjuvant treatment such as ADT (androgen deprivation therapy) or radiotherapy to avoid relapse. These treatment strategies mostly depend on the risk stratification of the patient 6, 7. PRAD is a highly heterogenous disease with enriched stromal content in tumor 8. Increasing evidences point out that the tumor microenvironment plays an important role in initiation and progression. Chronic inflammation is prevalent in the adult prostate and serves as a probable risk factor for initiation of prostate cancer, however, the complex mechanism is poorly understood 9. Furthermore, current data indicates that prostate cancer benefits less from immunotherapy 10. The low tumor mutation burden, lack of tumor antigen, or lack of immune checkpoint covered by the current immune checkpoint inhibitor, lack of immune cell infiltration can be reasons accounting for the low response to immunotherapy 11. Therefore, focusing on the tumor microenvironment (TME) of PRAD might provide some clues for solving the problems above.

Neutrophil cells have emerged as an important component of the TME. Neutrophil extracellular traps (NETs) are web-like filamentous extracellular structures formed by neutrophil, consists of DNA, histones and cytotoxic granule-derived proteins. They can have dual functions, including tumor-promoting and tumor-suppressing effects via exerting effects on tumor cells and non-tumor cells 12. NETs components promote the antigen-presenting process by activating dendritic cells (DCs) through TLRs 13 or being internalized by fibroblasts which enhance surface MHC upregulation 14. Furthermore, NETs have ability to degrade the cartilage matrix which exposing the cartilage antigens to T cells 15. Additionally, B cells could also be activated by NETs components 16. And T helper (Th) cell differentiation could also be modulated by NETs 17. Recent study demonstrated the activation of DCs by NETs indeed induce CD8^+^T cell responses against tumor cells 18 and NETs promote T cell priming by reducing their activation threshold 19. In pancreatic cancer, IL17-induced NETs formation mediate resistance to checkpoint blockade by exclusion and inactivation of CD8^+^ T cells 20. NETs can act as a physical barrier to limit contact between cancer cells and cytotoxic natural killer or T cells 21, 22. PD-L1 embedded within NETs can also cause T cell exhaustion to modulate the TME 23. NETs can stimulate the CD4^+^T cell differentiation into regulatory T cells via TLR4 signaling to promote cancer development 24. IL17 secreted by γδ T cells induced formation of NETs, which suppressed CD8^+^T cells recruitment to tumor 20. For tumor cells, NETs have been reported to promote the tumor angiogenesis via activating the AKT/mTOR signaling in endothelial cell in gastric cancer 25. The chemotherapy-induced NETs formation reduces therapy efficacy against breast cancer lung metastasis by activating the TGF-β-dependent epithelial-mesenchymal transition (EMT) in cancer cells 26. NETs formation induced by a tumor-secreted protease, CTSC, promote lung colonization of breast cancer 27. To sum up, NETs mediate its tumor-promoting or tumor-suppressing effect via various mechanisms. NETs-targeted therapy might be potential for enhancing the efficacy of current therapy. However, there is lack of NETs related study in PRAD to date. In this study, we try to delineate the role of NETs in PRAD.

## Material and methods

### Data collection

RNA-seq data were obtained from TCGA. R package “TCGAbiolinks (version 2.25.0)”^28^ was used to download the gene expression profiles, clinical information and copy number variation from TCGA-PRAD dataset. R package “VarScan2 Variant Aggregation and Masking” was used to predict the single nucleotide variation (SNV). There are 484 tumor samples and 51 adjacent normal samples in the TCGA-PRAD dataset, among them 416 tumor samples with complete clinical information were included for further analysis. The 312 NETs-related genes were collected from previous studies^29^-^33^ (**Table S1**).

### Analysis of NETs (Neutrophil Extracellular Traps) related genes with differential expression

R package “limma (version 3.50.0)” 34 was used to identify the differential-expressed genes (DEGs) between the adjacent normal samples (N=51) and tumor samples (N=484). The overlap genes between DEGs and 312 NETs-related genes were obtained for further analysis. The amplification and deletion of NETs related genes in PRAD were shown. Somatic mutation, the genetic locus, and copy number variation (CNV) of 19 NETs related genes were analyzed.

### Functional enrichment analysis

NETs-related genes with different expressions were selected for further analysis. GeneMANIA was used to construct the protein-protein interaction network. We employed Gene Ontology (GO) and Kyoto Encyclopedia of Genes and Genomes (KEGG) analyses with R package “clusterProfiler” to explore the NETs-related genes with different expressions. GSEA was performed to identify the difference of signaling pathway and biological effects between the identified subtypes.

### Consensus clustering analysis based on NETs related genes

We conducted consensus clustering analysis with the R package “ConsensusClusterPlus” to identify the NETs-related molecular subtypes 35. Cluster numbers were set from 2 to 6 and replicated process repeated 1,000 times. Survival analysis was estimated by the Kaplan-Meier method and the differences were examined by the log-rank test.

### Genes Set Variation Analysis (GSVA)

To evaluate the biological difference of different subtypes, the “c2.cp.kegg.v7.5.1.symbols” was retrieved from MSigDB database as the reference gene sets. R package “GSVA (version 142.0)” was used to perform gene set variation analysis between different PRAD subtypes. R package “limma” was used to compared the GSVA scores of each gene set between different PRAD subtypes. Moreover, 50 hallmark gene sets were downloaded from MSigDB database as reference gene set, the enrichment score of each gene set for each PRAD patient was then estimated using the GSVA method in the R package "GSVA".

### Immune infiltration analysis

ssGSEA was used to evaluate the dysregulation of specific gene set for each sample. We calculated the level of infiltrating immune cells of each case based on the gene expression profiles. R package “ggplot2” were used to visualize the difference of infiltrating immune cells between each cluster.

### Construction and validation of prognostic model

We further investigated the prognostic value of the differential-expressed NETs-related genes. We identified the differential-expressed genes between the PRAD subtypes discerned based on the differential-expressed NETs-related genes. All tumor samples with complete clinical information were randomly grouped into training set (n=299) and validation set (n=117). LASSO cox regression analysis was performed to construct the prognostic model. Risk score was calculated as following:

Risk score=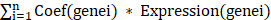
(Coef (genei): coefficient, Expression (genei): gene expression level)

We then separated PRAD patients into low- and high-risk group with the median risk score value as cut-off. The survival curve of low- and high-risk group were generated with Kaplan-Meier method and the differences were examined by the log-rank test. ROC analyses were performed to evaluate the performance of the prognostic model.

### Construction and validation of nomograph

Cox uni-variable analysis and multi-variables analysis were conducted to identify the significant survival-related signature. R package “RMS” was used to predict the survival of 1 year, 3 years and 5 years respectively. ROC analysis was performed to evaluate the nomograph.

### Drug sensitivity analysis

R package oncoPredict (version 0.2) was used to investigate the sensitivity of chemotherapeutic agents and targeted drugs between the high-risk group and low-risk group 36. We combined the gene expression data of cell lines from The Genomics of Drug Sensitivity in Cancer Project dataset (GDSC) with expression profiles from TCGA samples.

### Statistics analysis

All statistical analyses were performed with R software (version 4.2.1). Spearman's rank correlation analysis was performed to evaluate the correlations between two continuous variables. Wilcoxon rank sum test was performed to evaluate the difference between two groups. Kruskal-Wallis Test was performed to evaluate the difference among three groups or more than three groups. The threshold for statistical significance was set as* P* < 0.05.

## Results

### Expression and mutation landscape of neutrophil extracellular traps related genes in PRAD

**Figure 1:**
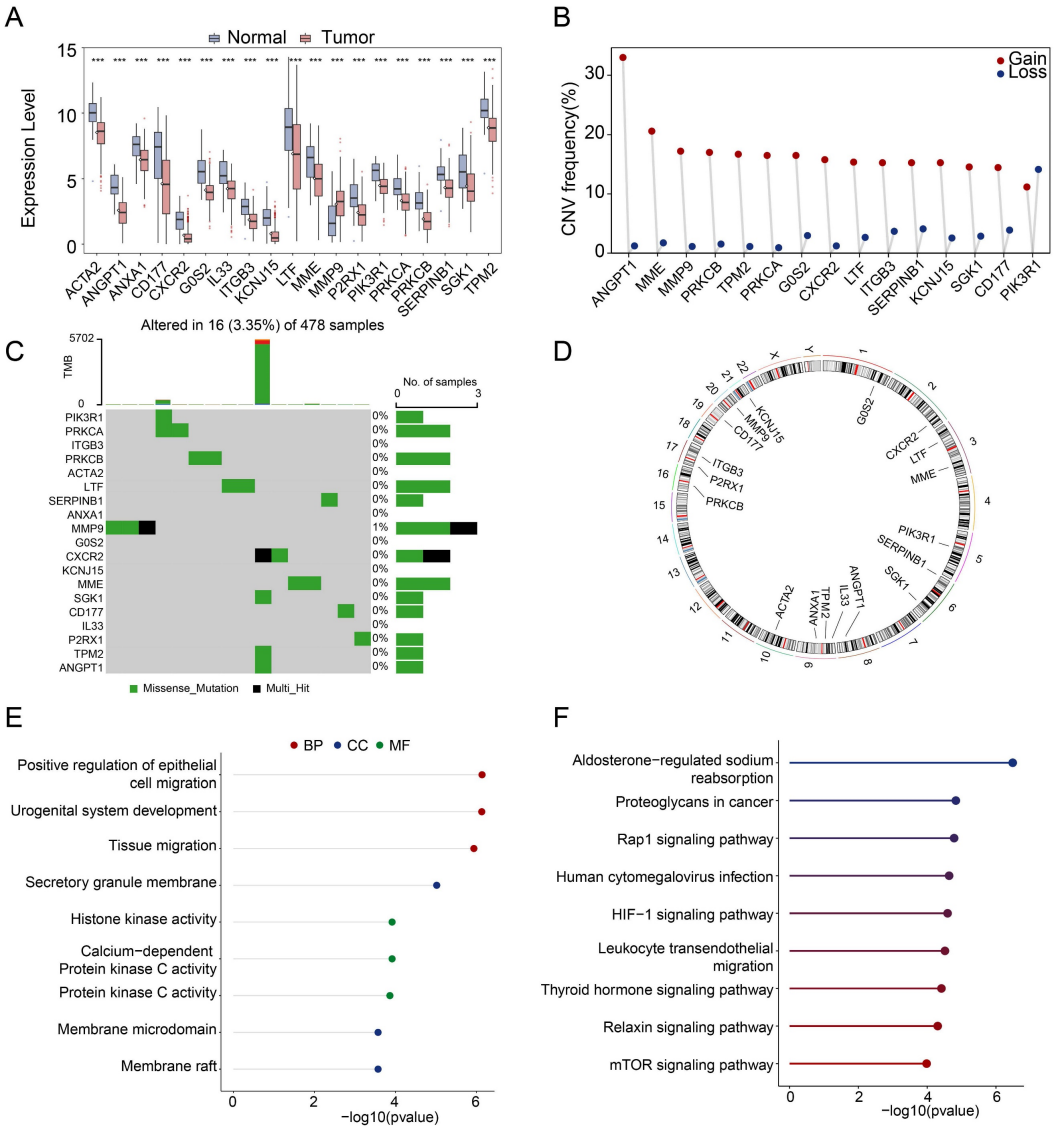
** Identification and functional annotation of differential-expressed NETs-related genes in PRAD. (A)** A barplot showing the 19 differential-expressed NETs-related genes in PRAD, ***, *P* < 0.001 of Wilcoxon test. (**B-D**) The landscape of copy number variation (**B**), somatic mutation landscape (**C**) and chromosomal position (**D**) of 19 differential-expressed NETs-related genes in PRAD. (**E-F**) GO analysis (**E**) and KEGG pathway analysis (**F**) of the 19 differential-expressed NETs related genes.

The workflow of this study was shown in **Figure S1**. Compared with adjacent normal samples, a total of 1221 genes were differential-expressed, including 397 being upregulated and 824 being downregulated in PRAD (**Figure S2A**). To identify the differentially-expressed NETs-related genes, we constructed a NETs-related gene set consisting of 312 genes based on previous study 29-33 (**Table S1**), 19 NETs-related genes were identified with different expressions including 18 genes being downregulated and 1 gene being upregulated in PRAD (**Figure S2A**). To be more specific, ACTA2, ANGPT1, ANXA1, CD177, CXCR2, G0S2, IL33, ITGB3, KCNJ15, LTF, MME, P2RX1, PIK3R1, PRKCA, PRKCB, SERPINB1, SGK1, TPM2 were downregulated, while MMP9 was upregulated (**Figure 1A**). Among these 19 differential-expressed NETs-related genes, most of them had gain of copy number (**Figure 1B**). The somatic mutation and chromosomal location of these 19 genes were also analyzed (**Figure 1C, D**).

### Function enrichment analysis of the differential-expressed NETs-related genes

GO and KEGG pathway analyses were performed based on the 19 differential-expressed NETs-related genes. As a result, these genes were involved in biological processes such as positive regulation of epithelial cell migration, urogenital system development and tissue migration in GO analysis (**Figure 1E**). KEGG pathway analysis revealed that these genes were mainly associated with Aldosterone-regulated sodium reabsorption, Proteoglycans in cancer, Rap1 signaling pathway, Human cytomegalovirus infection, HIF-1 signaling pathway, Leukocyte transendothelial migration, Thyroid hormone signaling pathway, Relaxin signaling pathway and mTOR signaling pathway (**Figure 1F**). We then constructed a protein-protein interaction (PPI) network to further delineate the connections between these NETs-related genes (**Figure S2B**).

### Consensus clustering identified two subtypes of PRAD

**Figure 2:**
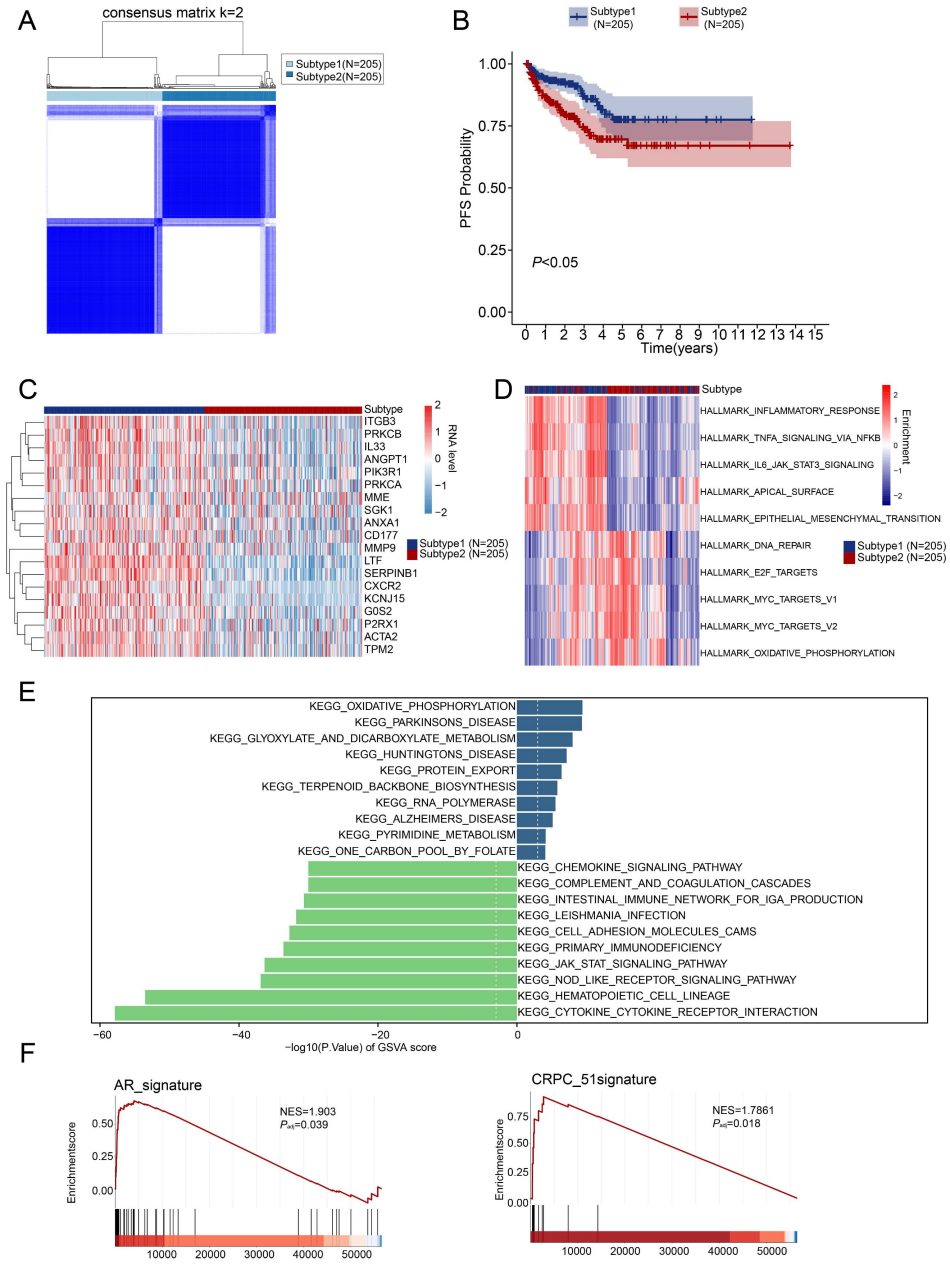
** Identification of NETs-related subtypes.** (**A**) Two subtypes of PRAD were identified by consensus clustering. (**B**) Progression-free survival analysis of the two PRAD subtypes. (**C**) Heatmap revealed the expression pattern of the 19 NETs-related genes in two PRAD subtypes. (**D-E**) Hallmark analysis (**D**) and KEGG (**E**) pathway analysis of the two PRAD subtypes. (**F**) Androgen receptor signaling pathway (AR signaling) (*left panel*) and signature related to intrinsic androgen deprivation therapy (ADT) resistance were activated in subtype 2 (*right panel*).

Consensus clustering analysis based on the 19 differential-expressed NETs-related genes determined two subtypes of TCGA-PRAD patients, namely subtype 1 and subtype 2 (**Figure 2A**). Subtype 1 has better progression-free survival rate (**Figure 2B**). Moreover, the OS (Overall Survival), BCR-free survival (Biochemical Recurrence Free Survival) or DFS (Disease Free Survival) was not significantly different between subtypes, but there were tendencies showing that subtype 2 had worse OS, BCR-free survival and DFS compared to subtype 1 (**Figure S2C**).

Heatmap was showing the expression of 19 differential-expressed NETs-related genes in two PRAD subtypes, and subtype 1 showed higher expression of the 19 NETs-related genes compared with subtype 2 (**Figure 2C**). We then explored the key signal pathways and biological effects in each subtype, which might clarify molecular mechanism that leading to different clinical outcomes. Hallmark analysis showed that subtype 1 has activation in inflammatory response, TNFA-NFKB signaling, IL6-JAK/STAT3 and EMT while subtype 2 has activation of DNA repair, E2F target, MYC target and oxidative phosphorylation (**Figure 2D**). GSVA analysis of the two subtypes pointed out that cytokine-cytokine receptor interaction was activated in subtype 1 while subtype 2 exerted activation of oxidative phosphorylation (**Figure 2E**). Additionally, we also observed the activation of the Androgen receptor (AR) signaling pathway and the CRPC_51 signature consists of 51 genes that were significantly upregulated in CRPC (Castration-resistant Prostate Cancer)-like cells in hormone-naïve early PRAD 37 was also enriched in subtype 2 (**Figure 2F**).

### Landscape of somatic mutations and immune infiltration in different NETs related subtypes

**Figure 3:**
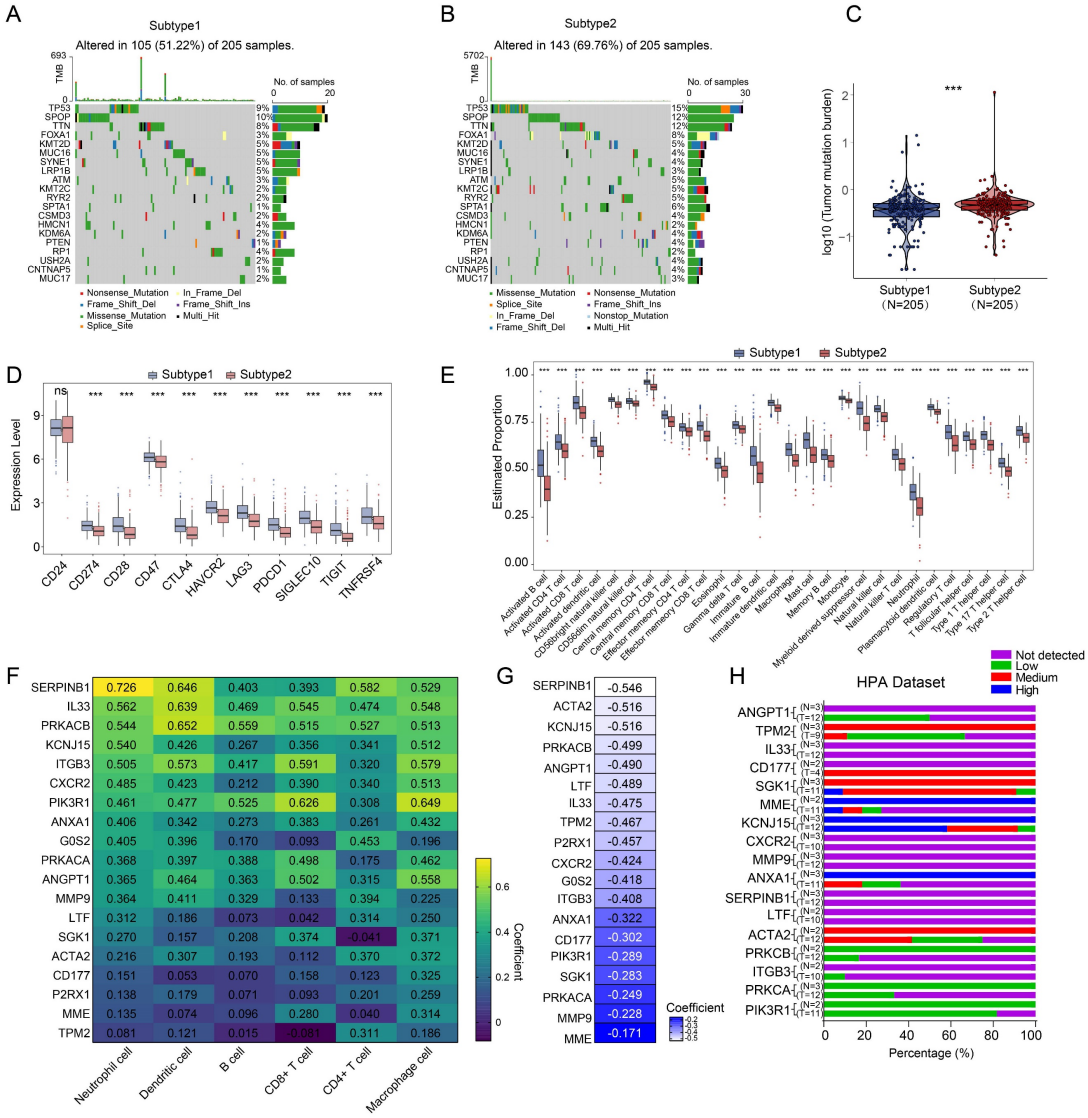
** Somatic mutation and tumor microenvironment landscape in different subtypes.** (**A-B**) The somatic mutation profiles in subtype 1 (**A**) and subtype 2 (**B**). (**C**) Shown were tumor mutation burden of subtype 1 and subtype 2. (**D**) Expression of immune checkpoints in subtypes of PRAD. (**E**) Difference of immune cell infiltration in subtypes of PRAD. (**F**) Correlation analysis between immune cell infiltration and expression level of 19 NETs related genes retrieved from the TIMER database. (**G**) Correlation analysis between tumor purity and expression level of 19 NETs related genes retrieved from the TIMER database. (**H**) Protein expression data in normal prostate tissues and prostate cancer tissues detected by Immunohistochemistry (IHC) were retrieved from Human Protein Atlas (HPA) dataset. ***, *P* < 0.001; ns, not significant of Wilcoxon test in this figure.

For further analysis, we selected the 410 tumor samples with complete survival information, among them, 248 samples were identified with the top 20 most frequent mutations. Subtype 1, exerted a lower somatic mutation frequency compared to subtype 2 (**Figure 3A, B**). Among them, TP53 (9% vs 15%), SPOP (10% vs 12%) and TTN (8% vs 12%) ranked the top three most frequent genes. Subtype 2 also showed higher overall tumor mutation burden (**Figure 3C**). Moreover, most immune checkpoints exerted higher expression in subtype 1 (**Figure 3D**) and significant differences were also observed in the level of 28 types of immune cells (**Figure 3E**). The expressions of the 19 NETs-related genes were positively correlated with immune cell infiltration (**Figure 3F**) but negatively correlated with the tumor purity according to TIMER database 38 (**Figure 3G**). Additionally, we successfully retrieved the protein expression data of 17 NETs-related genes, while the data for G0S2 and P2RX1 were not available. Most of them were low-expressed in both the normal prostate glandular cells and prostate cancer cells according to the Human Protein Atlas (HPA dataset) (**Figure 3H**). Moreover, the PRAD single cell expression data retrieved from TISCH database 39 also supported that most of the 19 NETs related genes were mostly expressed in immune cells or stromal cells rather than tumor cells (**Figure S3**). These data indicated that the identified NETs related genes might be mostly expressed in varied stromal cells or immune cells to exert effect on the TME.

### Prognostic significance of neutrophil extracellular trap-related genes in PRAD

**Figure 4:**
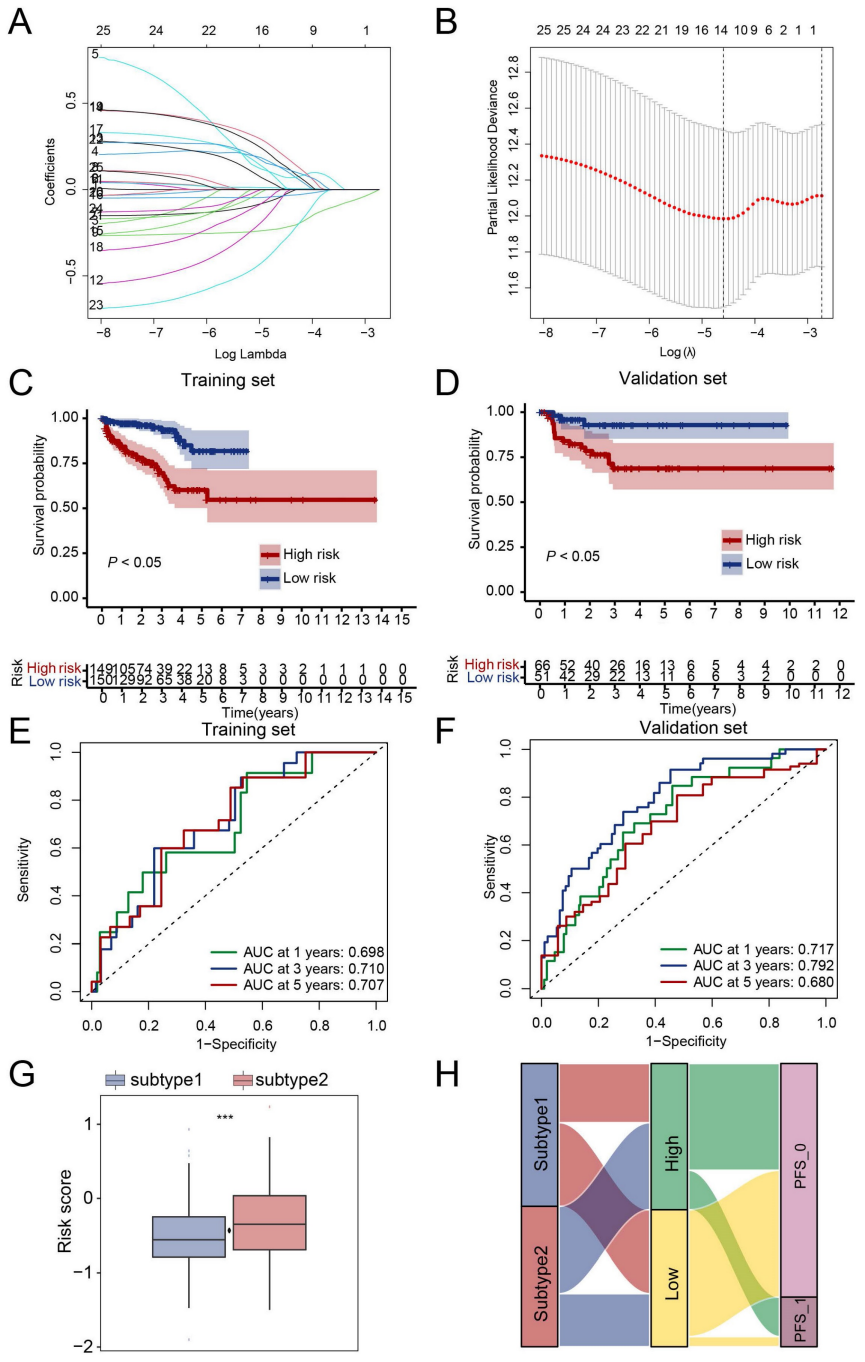
** Prognostic signature in PRAD based on NET-related genes in TCGA dataset.** (**A-B**) The coefficient and partial likelihood deviance of the prognostic signature analyzed by LASSO analysis. (**C-D**) PRAD patients in the high-risk group had a poor PFS rate compared to those in the low-risk group in both training set (**C**) and validation set (**D**). (**E-F**) ROC curve of the risk score for predicting 1-year, 3-year, and 5-year PFS in both training set (**E**) and validation set (**F**). (**G**) Risk score comparison between subtypes, ***, *P* < 0.001 of Wilcoxon test. (**H**) Correlation of NETs-based subtypes and groups identified by risk score. PFS_0, without progression at the time point of follow-up; PFS_1, with progression at the time point of follow-up.

Next, we tried to evaluate the clinical value of the NETs-based subtype. Firstly, 388 genes with different expressions between the two subtypes were selected. Secondly, LASSO analysis was performed and 13 candidate genes CYBA, CORO1A, LTF, LCN2, MMP9, IL1B, IL6, CCL2, CD177, CFTR, CXCL2, S100A9, SOCS3 (**Table S2**) were selected according to the coefficients and least partial likelihood deviance to further construct the prognostic model (**Figure 4A, B**). The risk score of each PRAD case was calculated based on the 13 genes and the PRAD cases were divided into two groups based on the risk score, patients in the high-risk group had a poor PFS compared with those in the low-risk group in both training set and validation set (**Figure 4C, D**). Moreover, the AUC analyses of 1-year, 3-year, and 5-year Progress-Free survival were 0.698, 0.710, 0.707 and 0.717, 0.792, 0.680 in the training set and validation set respectively (**Figure 4E, F**). The risk score of subtype 2 were higher than subtype 1 (**Figure 4G**). We further evaluated the patient distribution across the two NETs subtypes and two risks core groups (**Figure 4H**). Moreover, univariate cox regression analysis revealed that NETs-related risk score, subtype, lymph node status, pathological tumor stage and clinical tumor stage were independent prognostic factors for PRAD patients (**Figure S4A**). After taking the above factors into multivariate cox regression analysis, the result revealed that the risk score was still an independent predictive factor (**Figure S4B**). A nomogram including risk score, pathological tumor stage and clinical tumor stage was created to predict 1-year, 3-year, and 5-year PFS rates (**Figure S4C**). The AUC analyses on the nomogram model of 1-year, 3-year, and 5-year survival achieved 0.738, 0.765, 0.730 respectively, showing improved accuracy (**Figure S4D**).

### MMP9 might be a key regulator of NETs in PRAD

**Figure 5:**
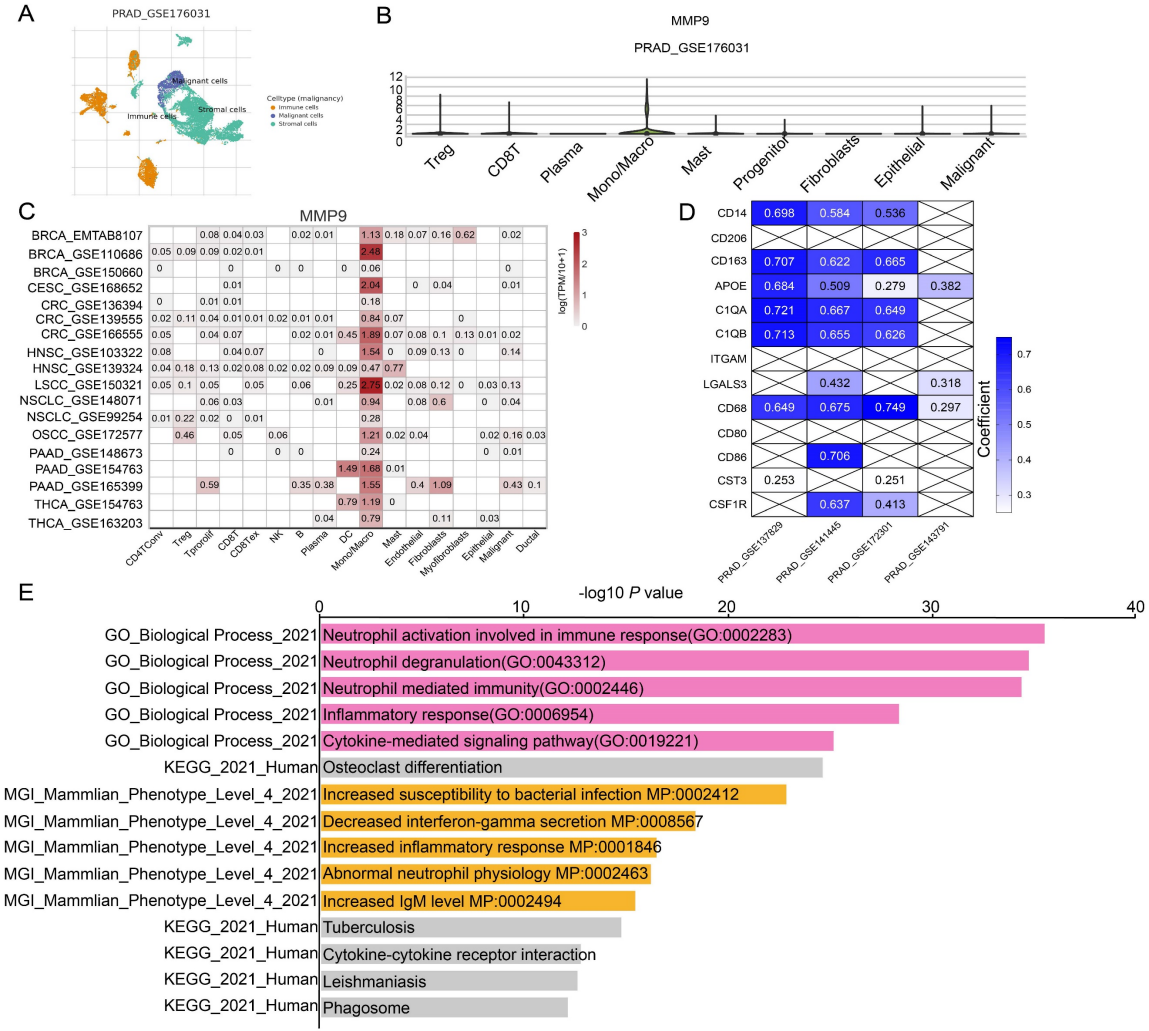
** Identification of MMP9 as a key NETs regulator in PRAD.** (**A**) Uniform Manifold Approximation and Projection (UMAP) plot showed the cell clusters in GSE176031 dataset. (**B**) Violin plot showed the distribution of MMP9 across different cell clusters in tumor tissue in GSE176031 dataset. (**C**) Heatmap showing the expression levels of MMP9 in single-cell RNA-seq datasets of other cancer types from TISCH database. (**D**) Correlation among MMP9 and classic Mono/Macrophage cell markers in PRAD single-cell RNA-seq datasets. (**E**) Functional annotation of the 291 MMP9 correlated genes from Enrichr web resource.

Next, we tried to find the key regulator driving the NETs subtype in PRAD. We focused on MMP9, LTF, CD177, as these three genes were not only included in 19 NETs-related genes with differential expressions, but also selected for prognostic signature construction by LASSO analysis. We found that MMP9 was mostly expressed in Monocyte/Macrophage cells according to multiple PRAD single-cell datasets in TISCH database (**Figure 5A-B, Figure S3P, Figure S5A**), while the expression patterns of LTF and CD177 were varied (**Figure S3Q, S**).

Moreover, MMP9 was also tend to be expressed in Mono/Macrophage cell in other cancer types (**Figure 5C**). Moreover, MMP9 expression was positively correlated with expression of most Mono/Macrophage cell markers demonstrated by both the single-cell dataset (**Figure 5D**) and the TCGA-PRAD dataset (**Figure S5B**). Furthermore, we retrieved 921 and 1088 genes whose expressions correlated with MMP9 expression by ULCAN 40 and TISCH database based on the TCGA-PRAD dataset and single-cell dataset respectively, and 291 genes were identified by overlap analysis (**Figure S5C**). Interesting, functional analysis by Enrichr 41 pointed out that the 291 MMP9-correlated genes were involved in biological process related to formation of NETs, including neutrophil activation and neutrophil degranulation by GO analysis. The cytokine-cytokine receptor interaction was also enriched by KEGG analysis.

Additionally, the 291 genes were correlated with mammalian phenotypes such as decreased interferon-gamma secretion, increased inflammatory response and abnormal neutrophil physiology by Mouse Genome Informatics (**Figure 5E, Figure S5D**). Furthermore, similar conclusions were driven in CRC (Colorectal cancer), HNSC (Head and Neck squamous cell carcinoma), PAAD (Pancreatic adenocarcinoma) and NSCLC (Non-small cell lung cancer) as concluded from PRAD, as genes highly correlated with MMP9 were enriched in pathway related to NETs such as Neutrophil degranulation and Neutrophil activation involved in immune response. However, the MMP9 correlated genes were not enriched in pathway related to NETs formation when analyzing the BRCA (Breast invasive carcinoma) or CESC (Cervical squamous cell carcinoma and endocervical adenocarcinoma) dataset (**Figure S6**). These data above implied that MMP9 expressed in Mono/Macrophage cells might exert effects on neutrophil leading to dysregulation of NETs.

### Potential therapeutic drugs prediction

**Figure 6:**
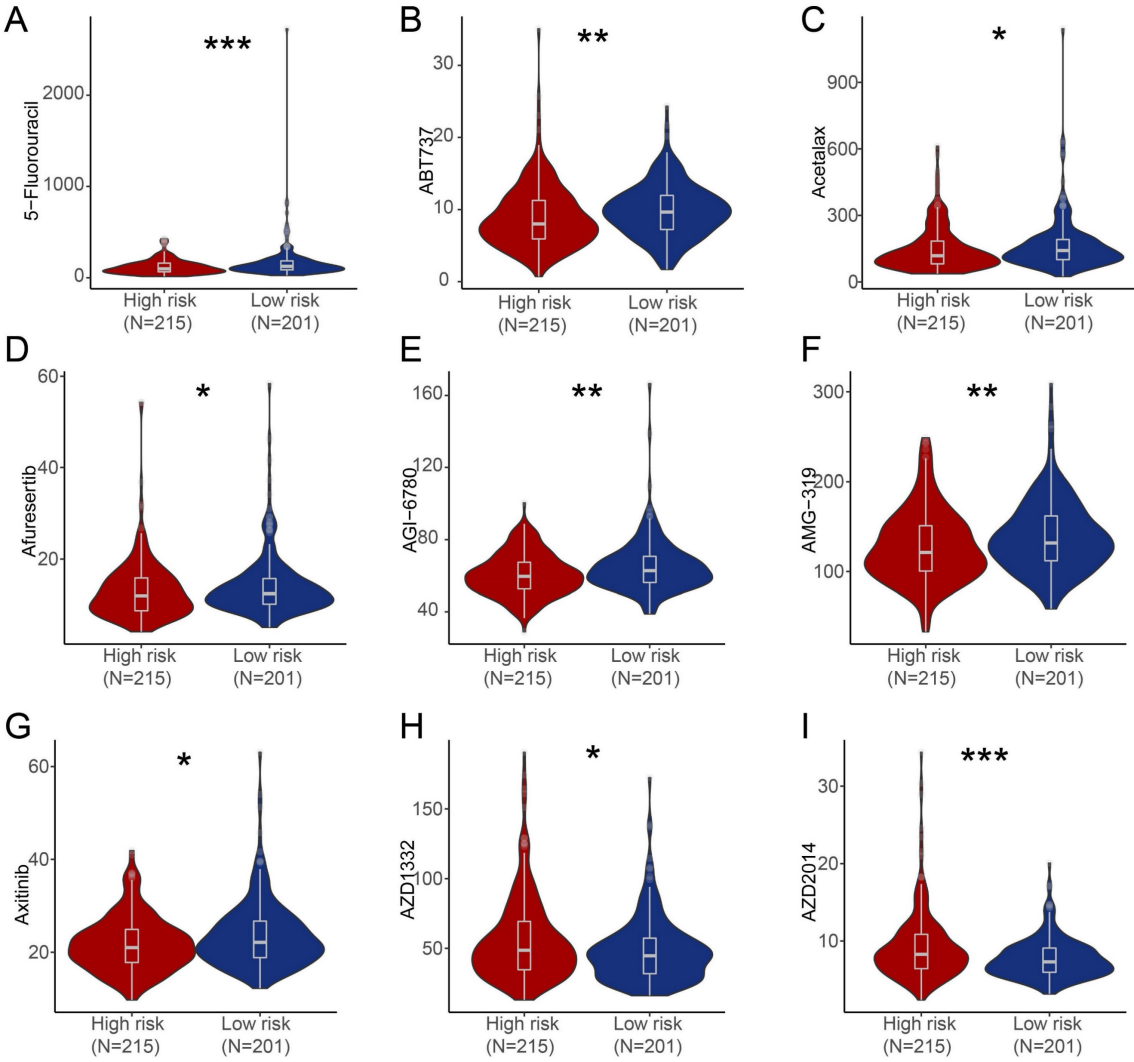
** Estimate of drug sensitivity in high-risk group and low-risk group of PRAD patients.** (**A-I**) Shown were predicted sensitivity of 5-Fluorouracil (**A**), ABT737 (**B**), Acetalax (**C**), Afuresertib (**D**), AGI-6780 (**E**), AMG-319 (**F**), Axitinib (**G**), AZD1332 (**H**), AZD2014 (**I**) in high-risk group and low-risk group of PRAD patients. *, *P* < 0.05; **, *P* < 0.01; ***, *P* < 0.001; ns, not significant of Wilcoxon test in this figure.

We further estimated the sensitivity of groups with different risk score to chemotherapeutic agents and targeted drugs by combining the gene expression patterns of cell lines from the Genomic of Drug Sensitivity in Cancer (GDSC) database. Patients in high-risk group were predicted to be more sensitive to 5-Fluorouracil, ABT737 (BCL-2 inhibitor), Acetalax, Afuresertib (AKT inhibitor), AGI-6780 (IDH inhibitor), AMG-319 (PI3K inhibitor), Axitinib (Tryosine kinase inhibitor) compared with patients in low-risk group, while patients in low-risk score group were predicted to be more sensitive to AZD1332 (NTRK inhibitor) and AZD2014 (mTOR inhibitor) than patients in high-risk group (**Figure 6**).

## Discussion

NETs are extracellular chromatin filaments formed by neutrophil, induced by cytokines such as IL8/CXCL8, CXCL1, CXCL2, G-CSF, cathepsin C, TLR ligands 21, 27, 42-45. Tumor-derived exosomes, cells within TME such as cancer-associated fibroblasts (CAFs) and platelets can also induce NETs. NETs play roles in infectious diseases 46, 47, cancer 48 and autoimmune diseases 49. Increasing studies pointed out that NETs play a significant role in cancer including tumor initiation 24, tumor dissemination 27, 50-52, chemoresistance 26 and modulating response to ICB (Immune Checkpoint Blockade) 20. As for prostate adenocarcinoma, once patients are diagnosed with PRAD, most patients will receive expectant therapy, localized resection or localized radiotherapy, and the ADT (androgen-deprivation therapy) will be launched after the surgery for patients who are defined as high risk for recurrence. The treatment strategies above are based on the risk stratification of patients. However, the current standard for risk stratification were mostly based on PSA level, clinical stage and Gleason pattern, both overtreatment and failure to detect patients with high risk for relapse exist 7. However, there is limited study on investigating the potential mechanism of NETs in PRAD. Thus, delineating the complex interplay between NETs and PRAD might provide valuable insights which are significant for optimizing the current therapeutic strategies.

In this study, we performed a systematic study regarding NETs-related genes in PRAD. We identified 19 NETs-related genes with differential expressions. Based on the expression pattern of NETs-related genes, two NETs related subtypes of PRAD were identified by consensus clustering, namely subtype 1 and subtype 2. Subtype 1 has better Progression-free survival rate than subtype 2. Subtype 1 has higher expression level of NETs-related genes than subtype 2, tend to be activated in hallmark such as inflammatory response, TNFA-NFKB signaling, IL6-JAK/STAT3. While the cell-cycle related pathway such as E2F1 targets and MYC targets which indicated worse prognosis 53, 54 were activated in subtype 2. Additionally, the AR signaling and signature indicating of intrinsic ADT resistance were also activated in subtype 2. The activation of these oncogenic pathways might account for the worse PFS for subtype 2 patients. In terms of tumor mutation burden, subtype 2 was higher than subtype 1. Further analysis revealed that subtype 1 has higher expression level of immune checkpoint and a higher abundance of immune cell infiltration, indicating an immune-exhausted status. As higher TMB are usually correlated with more infiltration of immune cell, which indicates sensitivity to immunotherapy. Tumor mutation burden (TMB) correlated with response to immunotherapy in solid cancers like melanoma, NSCLC but not for PRAD 55. PRAD seems to benefit less from immunotherapy according to the result of current clinical trials and there is a lack of effective biomarkers to predict the sensitivity of immunotherapy for PRAD patients 56. The NETs-based subtype signature might be potential biomarkers for predicting efficacy of immunotherapy. Based on the NETs-related subtypes, we further developed a prognostic signature. The PRAD patients with low-risk score had a favorable progression-free survival rate in both the training set and validation set. NETs-related risk score, clinical tumor stage and pathological tumor stage could act as independent prognosis factors for PRAD patients. Patients in high-risk group were predicted to be more sensitive to inhibitors target BCL-2, PI3K-Akt, IDH and Tyrosine Kinase compared to low-risk group, while patients in low-risk group were predicted to be sensitive to mTOR inhibitor and NTRK inhibitor compared to high-risk group.

At last, we identified MMP9 might be the key regulator of NETs in PRAD. MMP9 is one of the most abundant proteases of NETs. MMP9 (Matrix metalloproteinase 9) is member of the zinc metalloproteinase family of proteins that proteolyze the ECM and other substrates 57. There are many extensive studies revealed that MMP9 promoted tumor progression via modulating the TME. And more and more studies pointed out that MMP9 also regulated NETs. NETs associated MMP9 caused endothelial cell damage by cleaving endothelial pro-MMP2, which leaded to vascular dysfunction 58. On the other hand, NETs associated MMP9 can awake quiescent cancer cells by altering the ECM 50. It is reported that MMP9 is enriched in neutrophils, but the expression is more dynamic, for example, MMP9 is upregulated in obesity accompanied by more NETs formation, leading to impairment of the endothelial barrier 59. In this study, we identified that MMP9 as a NETs regulator in PRAD, mainly expressed in the Mono/Macrophage cells of PRAD, might exert influence on neutrophil activation which is related to NETs formation. In further study, we will focus on MMP9, delineating its mechanism in NETs formation in PRAD.

However, there are limitations of our study. Firstly, the expression of NETs related genes should be verified using clinical tissues, which will be presented in our further study. Secondly, the detailed mechanism of MMP9 in regulating NETs will be further verified by *in vivo* and *in vitro* study. Thirdly, the relationship between NETs-related subtypes and immune cell infiltration should be demonstrated via experiment.

## Conclusion

In conclusion, we performed a systematic study regarding NETs-related genes in PRAD. We identified 19 NETs-related genes with differential expressions and based on which distinguished the NETs-related subtypes with difference in terms of pathway activation, immune infiltration and tumor mutation burden. Furthermore, we built up a prognostic signature based on the NETs-related subtype by LASSO analysis. Further integrated analysis indicated that MMP9 expressed in Mono/Macrophage cells might mediate NETs formation via neutrophil activation in PRAD (**Figure S7**). These findings could provide more evidence for estimating prognosis and immunotherapy of PRAD patients.

## Supplementary Material

Supplementary figures and tables.

## Abbreviations

PRAD: Prostate adenocarcinoma
MMP9: Matrix Metalloproteinase 9
TME: Tumor microenvironment
MHC: Major Histocompatibility Complex
TLR: Toll-like receptors
ADT: Androgen deprivation therapy
TMB: Tumor mutation burden
CRC: Colorectal cancer
HNSC: Head and Neck squamous cell carcinoma
PAAD: Pancreatic adenocarcinoma
NSCLC: Non-small cell lung cancer
BRCA: Breast invasive carcinoma
CESC: Cervical squamous cell carcinoma and endocervical adenocarcinoma
NETs: Neutrophil Extracellular Traps
TCGA: The Cancer Genome Atlas
CAFs: Cancer-associated fibroblasts
ICB: Immune Checkpoint Blockade
OS: Overall Survival
BCR-free survival: Biochemical Recurrence Free Survival
DFS: Disease Free Survival
