# Supplementary material for: Identification of Molecular Subtype and Prognostic Signature for Prostate Adenocarcinoma based on Neutrophil Extracellular Traps
Source: J Cancer. 2024 Mar 17;15(9):2678–90. doi: 10.7150/jca.93275 (PMC10988314; doi:10.7150/jca.93275)
Supplement: Supplementary file 1 — Supplementary figures and tables. [file jcav15p2678s1.pdf]

Figure S1. The workflow of this study.

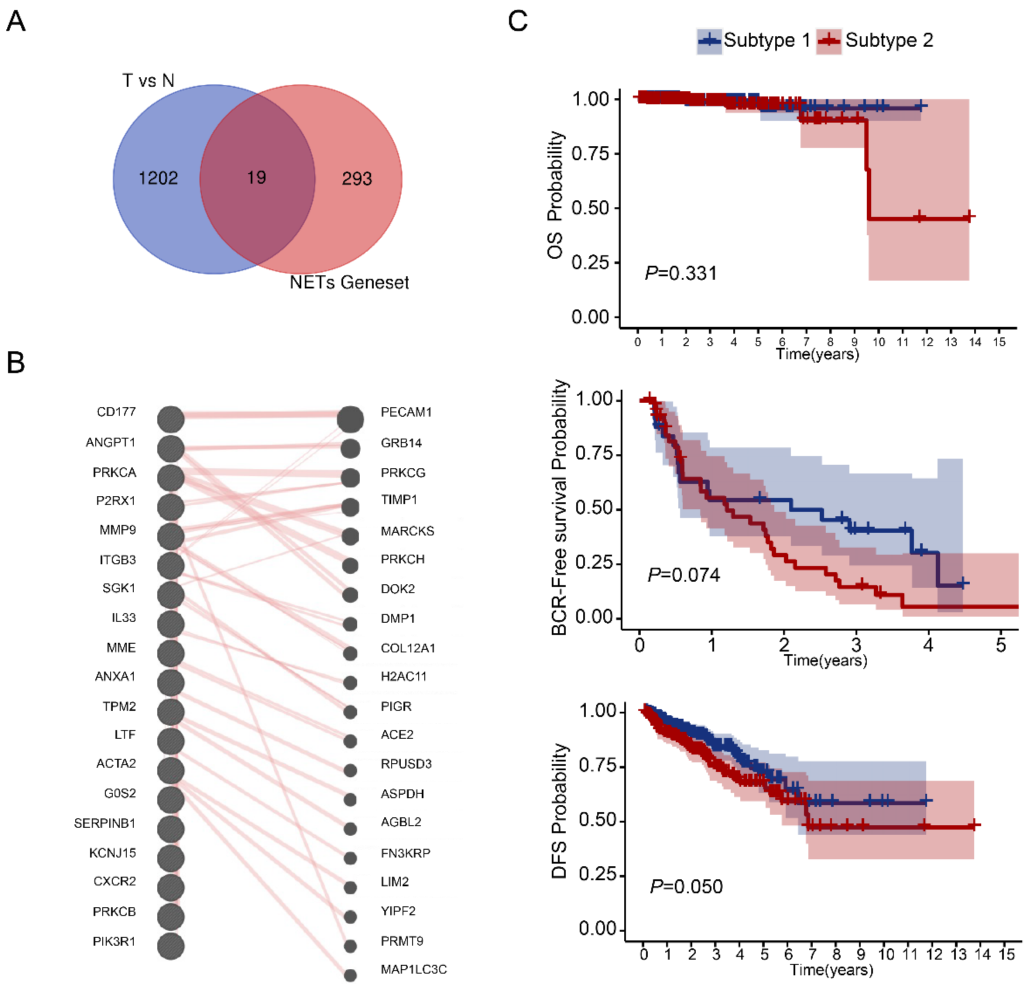

Figure S2. NETs related genes with differential expressions in PRAD and survival analysis of NETs related subtype. (A) Venn diagram about the number of differential-expressed NETs related genes between tumor (T) and adjacent normal tissue(N) in TCGA-PRAD dataset. (**B**) Protein-protein interaction analysis of the 19 differential-expressed NETs-related genes by GENEMANIA. (**C**) Overall survival (OS) analysis, Biochemical Recurrence Free survival (BCR-Free survival) and Disease-Free survival (DFS) analyses of the two PRAD subtypes.

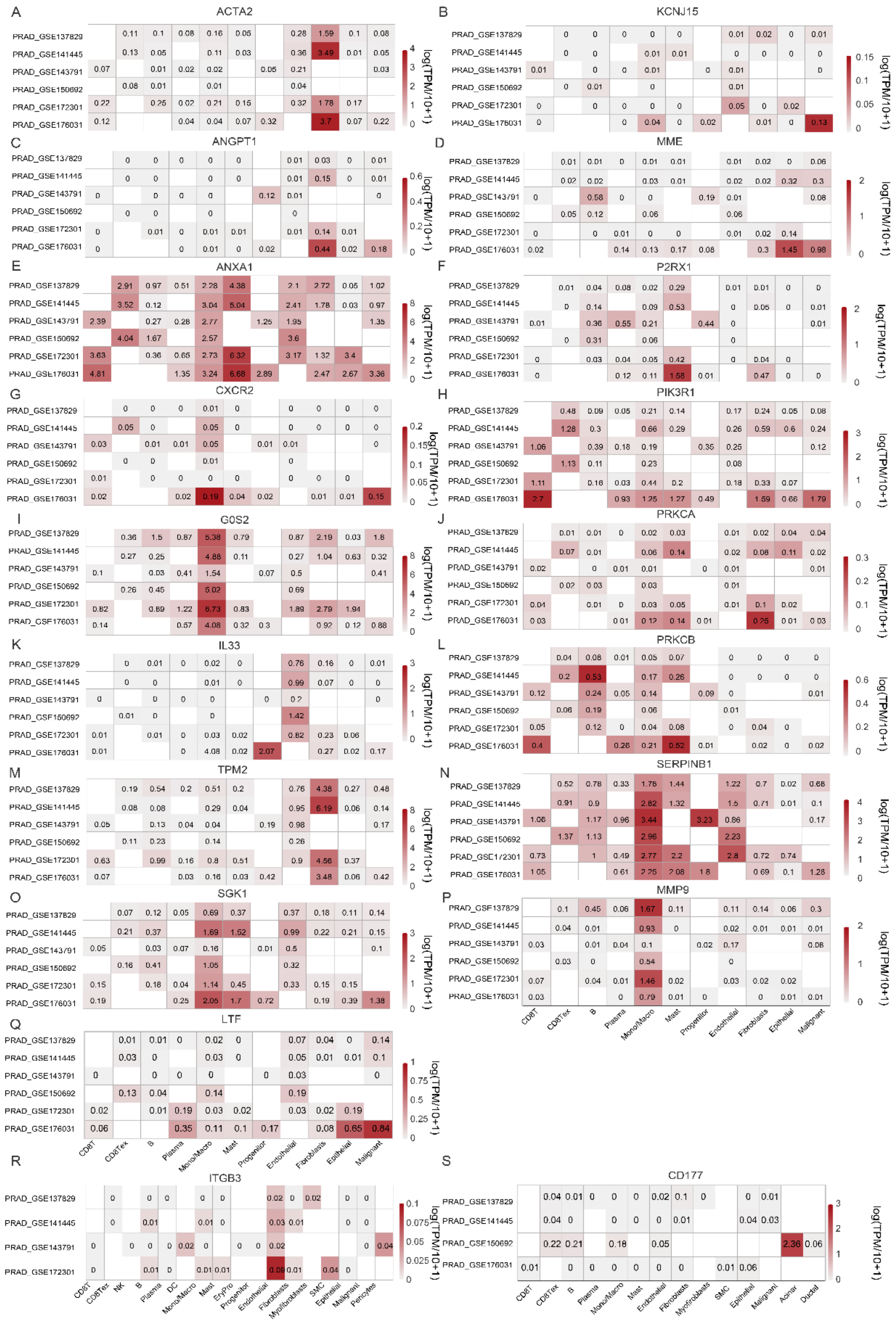

Figure S3 Heatmaps showing the expression levels of the 19 differential-expressed NETs related genes in PRAD single-cell RNA-seq datasets from TISCH database (**A-S**).

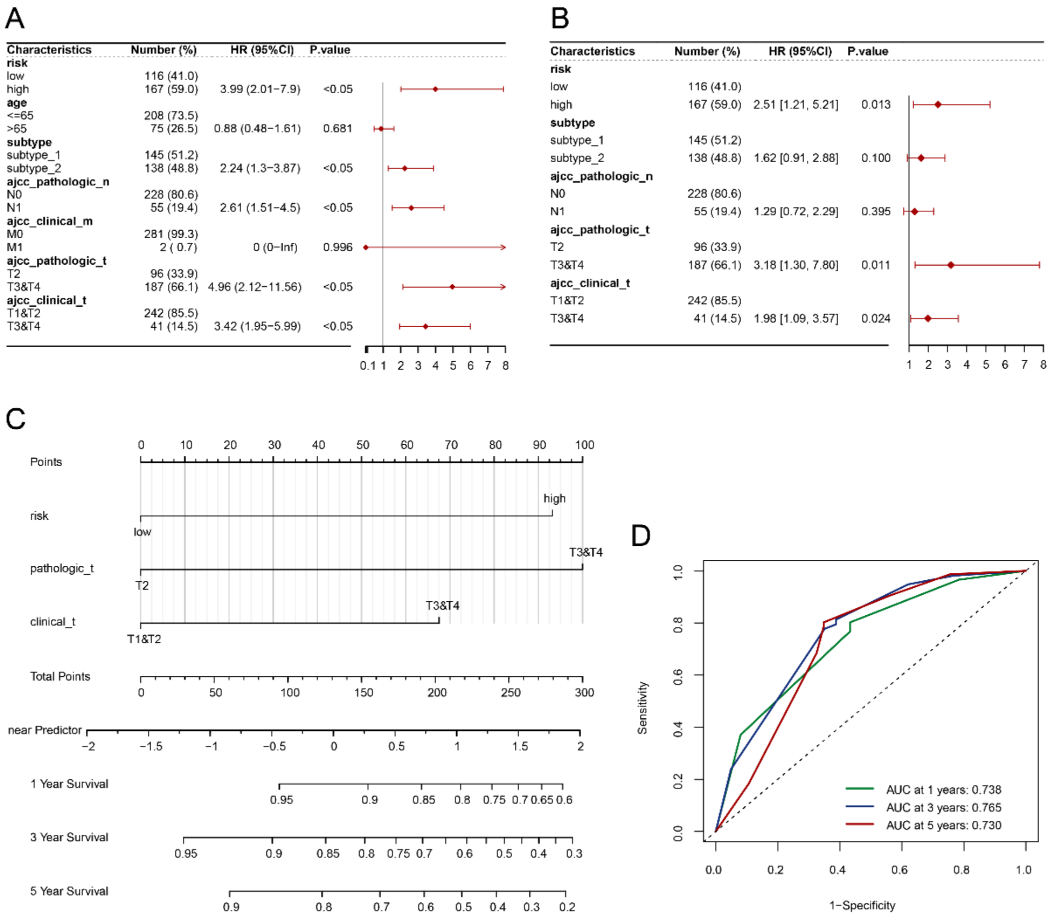

Figure S4. Development of a nomogram based on NETs-related risk score. (**A-B**) Univariate and multivariate cox regression analysis of clinical parameters and NETs-risk score. (**C**) A nomogram model for predicting the 1-year, 3-year, 5-year PFS of PRAD patients. (**D**) ROC curves of the nomogram for predicting 1-year, 3-year, and 5-year PFS.

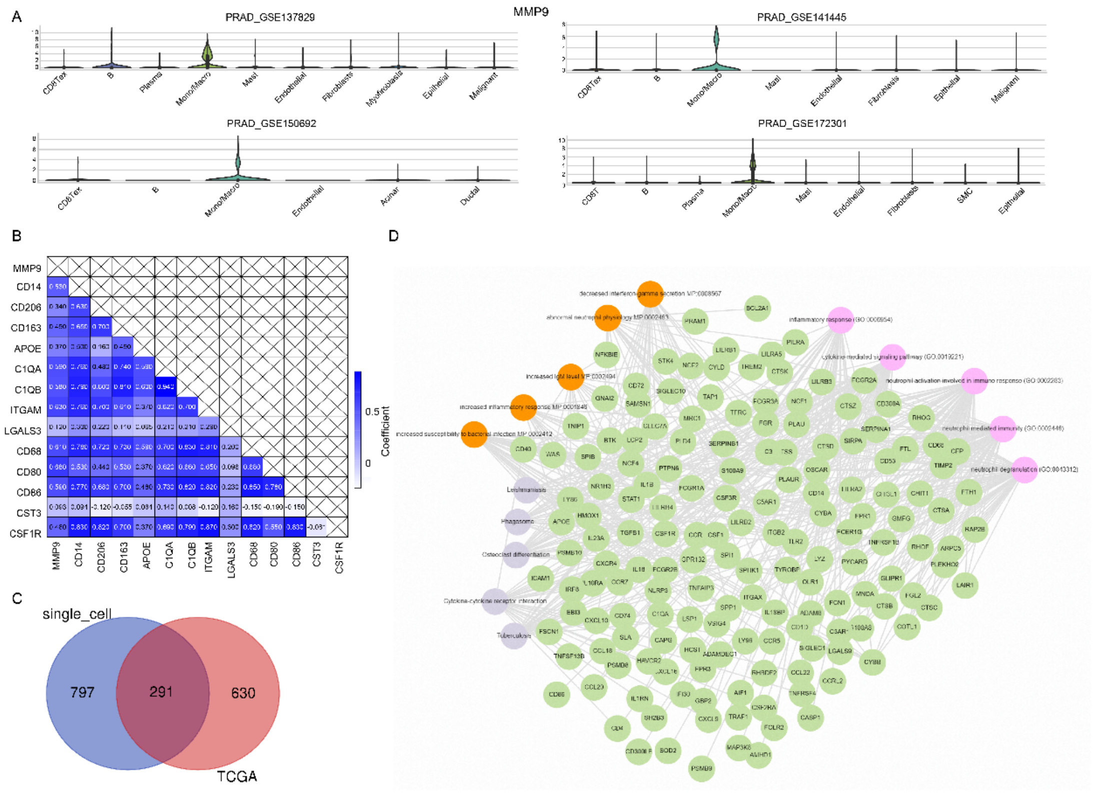

Figure S5. Identification of MMP9 as a NETs regulator in PRAD. (**A**) Violin plots showing the distribution of MMP9 across different cell clusters in tumor tissue in multiple PRAD single-cell datasets. (**B**) Correlation among MMP9 and classic Mono/Macrophage cell markers in TCGA-PRAD dataset. (**C**) 291 genes correlated with MMP9 were identified by both TCGA-PRAD dataset from ULCAN resource and PRAD single-cell datasets from TISCH database. (**D**) Network analysis of the 291 MMP9 related genes from Enrichr web resource.

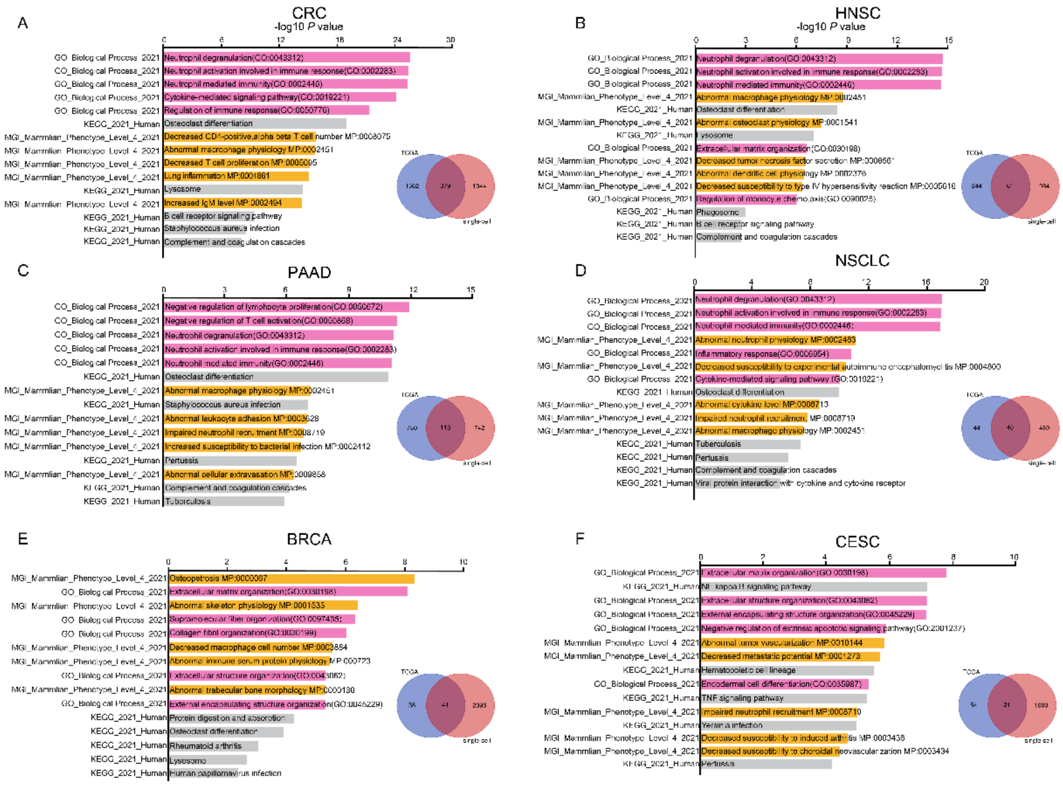

Figure S6 Functional annotation of the MMP9 correlated genes in other cancers.

Functional annotation of MMP9 correlated genes which indicated by both TCGA bulk RNA-seq data and single cell data for CRC (**A**), HNSC(**B**), PAAD (**C**), NSCLC (**D**), BRCA (**E**) and CESC (**F**).

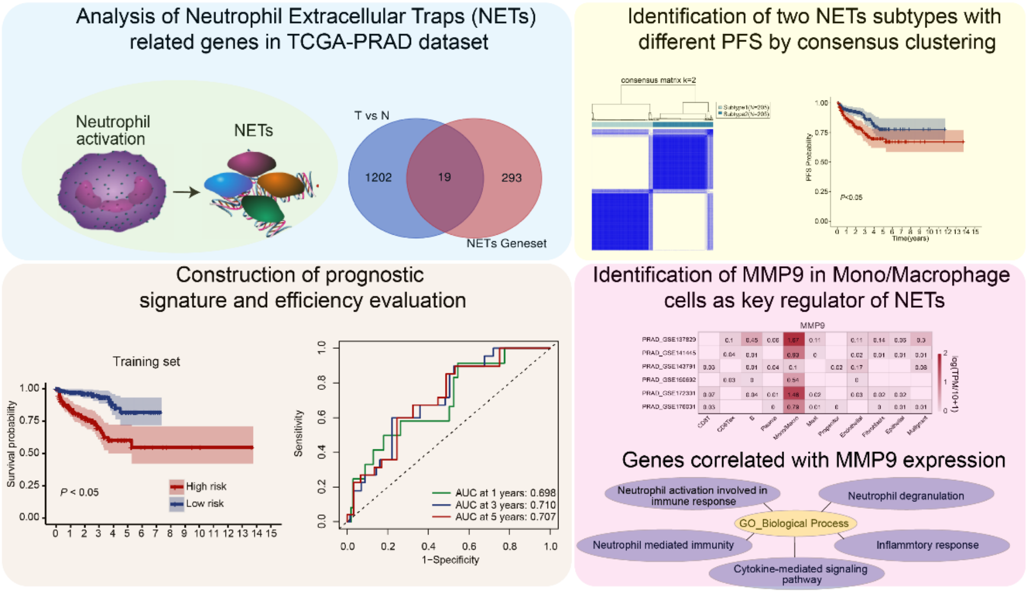

Figure S7. The main findings of this study.

Neutrophil extracellular traps (NETs) are extracellular structures formed by neutrophil, consists of DNA,

histones and cytotoxic granule-derived proteins. In this study, we identified 19 NETs related genes with differential expressions between PRAD and adjacent normal tissue. Two significant subtypes were identified based on these 19 genes by consensus cluster analysis, namely subtype 1 and subtype 2. Significant differences in prognosis were observed in these subtypes. LASSO Cox regression analysis identified a NETs-associated prognostic signature including 13 genes, and this signature had a good performance in predicting the progress-free survival of PRAD patients. Further analysis indicated that MMP9 mostly expressed in Mono/Macrophage cells might be key regulator of NETs formation via the neutrophil activation in PRAD.

Table S1 NETs geneset.

PIK3CA	PIK3R3	FPR1	AKT	SYK	FCGR3	MTOR	TLR2	HDAC3	VDAC3
PIK3CB	PRKCA	FPRL	RELA	PLCB	ITGB2	RAC2	TLR4	HDAC8	HDAC10
PIK3CD	VWF	RAF1	CLCN3	PLCG2	FCGR2A	CYBA	TLR8	HDAC4	PRKCB
PLCG1	FGA	MAP2K1	CLCN4	VDAC1	ITGA2B	NCF2	MPO	HDAC5	PRKCG
CTSG	FGB	MAP2K2	CLCN5	SLC25A4S	ITGB3	NCF1	HMGB1	HDAC6	AGER
ELANE	FGG	ERK	TLR7	SAP	SELP	NCF4	H2A	HDAC7	GSDMD
CASP1	C3	RAC1	ACTB	HDAC1	FCGR1A	ATG7	H2B	HDAC9	NOX2
NFKB1	C5	CASP4	ACTG1	HDAC2	SELPLG	PPIF	H3	HDAC11	AZU1
PIK3R1	C5AR1	MAP3K7	SRC	GP1BA	SIGLEC9	AQP9	H4	CAMP	SIGLEC14
PIK3R2	CR1	P38	ITGAL	ITGAM	IGH	CLEC7A	HAT1	VDAC2	SIGLEC5
CLEC6A	CTSC	MAPK1	GAPDH	ACTN4	HIST1H2AB	HSPA8	BST1	CPPED1	PDE4B
IRAK4	DEK	MAPK3	LDHB	MSN	HIST1H2BC	HSPA1A	CD93	FPR2	SLC22A4
RIPK1	PSGL1	TNF	LDHA	ACTR3	HIST1H3A	HSPA1L	CEACAM3	G0S2	SLC25A37

RIPK3	SIRL1	TALDO1	CORO1A	CAPZA1	LTF	MMP9	CREB5	HPSE	TECPR2
PIK3R5	IL10RA	TKT	ACTA2	HIST1H4A	SERPINB1	CAT	CRISPLD2	CXCR1	TNFRSF10C
AKT1	IL10RB	GPI	ACTN1	HIST1H2BA	LCN2	PRDX2	CSF3R	CXCR2	VNN3
AKT2	CSF1	ALDOA	PFN1	HIST1H2BB	LYZ	UBA52	CYP4F3	KCNJ15	DNASE1
AKT3	CSF2	TPI1	MYL6B	HIST3H3	ANXA1	S100A12	DYSF	LILRB2	ENTPD4
PAD4	CSF3	ENO1	ARPC1B	HIST1H2AA	ANXA3	RETN	FCAR	MGAM	F3
PADI4	CYBB	PGK1	GSN	HIST1H2BJ	PPIA	ALPL	FCGR3B	MME	IL17A
IL1B	CCL2	CXCL1	HIF1A	KLF2	MIR146A	NOX4	S100A9	WASL	HMGB2
IL6	CCL3	CXCL2	HRG	KRT10	MIR21	OPA1	S1PR2	XIST	TPM2
IL8	CCL4	CXCR4	IL12A	LCP1	MIR223	ORAI1	SOCS3	CXCL8	SH3BGRL3
KCNN3	CCL5	DEFA3	IL1RL1	LDLR	MNDA	P2RX1	SPP1	CXCL12	HMGN2
PTAFR	CD177	DNAJB1	IL33	LPAR3	MYD88	PARVB	STAT3	ARG1	HP1BP3
SGK1	CD274	F2RL2	IL36RN	MAPK14	MYH9	PF4	SUCNR1	HGF	ANGPT1
ARPIN	CD44	FCGR2B	IL5	MAPK7	NFE2L2	PKM	TICAM1	H2BC18	ANGPT2
C3AR1	CEBPB	FGL2	ILK	MCOLN3	NFIL3	PROCR	TIMP1	VIM	CGAS
CARD11	CFTR	GPBAR1	IRF1	MFN1	NFKBIA	PRTN3	TLR9	TMSB4X	STAT1
CCDC25	CLEC4E	H2AX	ITGB1	MFN2	NLRP3	S100A8	TNFAIP3	PGD	KRAS
CDC42	YAP1	VSTM1	MAP2K7	PRRT2	MOK	LTB4R2	FCGR3A	SULT2A1	LTB4R
TGFBR1	CCL17								

Table S2 13 NETs related genes used in prognostic model construction

CYBA	CORO1A	LTF	LCN2	MMP9	IL1B	IL6	CCL2	CD177	CFTR
CXCL2	S100A9	SOCS3							
